# Antidepressant-like effects of the *Punica granatum* and citalopram combination are associated with structural changes in dendritic spines of granule cells in the dentate gyrus of rats

**DOI:** 10.3389/fphar.2023.1211663

**Published:** 2023-10-13

**Authors:** Nelly-Maritza Vega-Rivera, María Eva González-Trujano, Alexandra Luna-Angula, Laura Sánchez-Chapul, Erika Estrada-Camarena

**Affiliations:** ^1^ Laboratorio de Neuropsicofarmacología, Dirección de Investigaciones en Neurociencias, Instituto Nacional de Psiquiatría “Ramón de la Fuente Muñiz”, Mexico City, Mexico; ^2^ Laboratorio de Neurofarmacología de Productos Naturales, Dirección de Investigaciones en Neurociencias, Instituto Nacional de Psiquiatría Ramón de la Fuente Muñiz, Mexico City, Mexico; ^3^ Laboratorio de Enfermedades Neuromusculares, División de Neurociencias Clínicas, Instituto Nacional de Rehabilitación “Luis Guillermo Ibarra Ibarra”, Mexico City, Mexico

**Keywords:** antidepressant-like effect, citalopram, dendritic complexity, *Punica granatum*, spinogenesis

## Abstract

**Introduction:** Natural products such as phytoestrogens-enriched foods or supplements have been considered as an alternative therapy to reduce depressive symptoms associated with menopause. It is known that the aqueous extract of *Punica granatum* (AE-PG) exerts antidepressant-like effects by activating β-estrogen receptors and facilitates the antidepressant response of the clinical drug citalopram (CIT). However, the effects on neuroplasticity are unknown. Objectvie investigated the antidepressant-like response of combining AE-PG and CIT at sub-optimal doses, analyzing their effects on the formation and maturation of dendrite spines in granule cells as well as on the dendrite complexity.

**Methods:** Ovariectomized Wistar rats (3-month-old) were randomly assigned to one of the following groups: A) control (saline solution as vehicle of CIT and AE-PG, B) AE-PG at a sub-threshold dose (vehicle of CIT plus AE-PG at 0.125 mg/kg), C) CIT at a sub-threshold dose (0.77 mg/kg plus vehicle of AE-PG), and D) a combination of CIT plus AE-PG (0.125 mg/kg and 0.77 mg/kg, respectively). All rats were treated intraperitoneally for 14 days. Antidepressant-like effects were evaluated using the force swimming test test (FST). The complexity of dendrites and the number and morphology of dendrite spines of neurons were assessed in the dentate gyrus after Golgi-Cox impregnation. The expressions of the mature brain-derived neurotrophic factor (mBDNF) in plasma and of mBDNF and synaptophysin in the hippocampus, as markers of synaptogenesis, were also determined.

**Results:** Administration of CIT combined with AE-PG, but not alone, induced a significant antidepressant-like effect in the FST with an increase in the dendritic complexity and the number of dendritic spines in the dentate gyrus (DG) of the hippocampus, revealed by the thin and stubby categories of neurons at the granular cell layer. At the same time, an increase of mBDNF and synaptophysin expression was observed in the hippocampus of rats that received the combination of AE-PG and CIT.

## Introduction

Estrogens are a factor in regulating emotions, mainly in women. Several studies have shown that hormonal fluctuations at some stages of women’s lives, such as the perimenopausal transition period, induce more anxiety and depressive symptoms (Woods et al., 2006; Bromberger et al., 2007; 2010; Joffe et al., 2007; Soares, 2010; 2014; Sander et al., 2021), and that estrogens are an effective treatment to alleviate depression symptoms associated with menopause (Rasgon et al., 2002; Cohen et al., 2003; Gordon and Girdler, 2014; Schmidt et al., 2015). In this sense, hormonal replacement therapy (HRT) induces beneficial effects on climacteric symptoms, like osteoporosis, hot flashes, and vasomotor symptoms. Evidence obtained from humans and rodents showed that HRT might improve the efficacy and shorten the latency to observe effects of antidepressants (Zanardi et al., 2007; Récamier-Carballo et al., 2012; Vega-Rivera et al., 2015; El-Khatib et al., 2020). However, other studies showed that using hormones as therapy could increase the risk of endometrial or breast cancer (Beral et al., 2011; Azam et al., 2018).

Research has been aimed at searching for new complementary therapies to improve depressive symptoms and decrease the side effects of estrogens. Several studies have suggested that natural alternatives such as foods or supplements with phytoestrogens can reduce menopausal symptoms (Cheng et al., 2007; Taku et al., 2010; 2012; Chen et al., 2015; Estrada-Camarena et al., 2017; Echeverria et al., 2021; Li et al., 2021) and exert antidepressant effects due to their estrogenic activity (Hu et al., 2017; Ko et al., 2020; Gorzkiewicz et al., 2021; Valdés-Sustaita et al., 2021; Park et al., 2022). Phytoestrogens are nonsteroidal natural compounds found in a wide variety of plants and foods that have a similar structure to estradiol (E2), with the capability to generate estrogenic or antiestrogenic effects through their binding to estrogen receptors (ER) (Morito et al., 2002; Kostelac et al., 2003; Zhao et al., 2011; Wang et al., 2020; Cho et al., 2021; Gorzkiewicz et al., 2021). Among the phytoestrogens with an important estrogenic-like activity are ellagitannins, which are mainly present in the *Punica granatum* (Pomegranate) (González-Trujano et al., 2015). The pomegranate has been recognized as a fruit with nutritional properties (Shaygannia et al., 2016), relevant anti-inflammatory (Adams et al., 2006; Rasheed et al., 2009), antioxidant (Gil et al., 2000; Mena et al., 2014; Les et al., 2015), anti-microbial (Saquib et al., 2021), anti-carcinogenic, anti-nociceptive, anti-depressive, and neuroprotective properties (Mori-Okamoto et al., 2004; Jurenka, 2008; Olajide et al., 2014; Velagapudi et al., 2016; Valdés-Sustaita et al., 2017; 2021). All these properties are related to the high content of phytochemicals, such as polyphenols, in all parts of the fruit (Wu and Tian, 2017; Bonesi et al., 2019; Shalaby et al., 2019; Ruan et al., 2022).

It is known that downregulation of neuroplasticity-related mechanisms, including dendritogenesis and synaptogenesis in specific brain areas, such as the hippocampus, may contribute to the pathophysiology of depression (Bessa et al., 2009; Castrén and Hen, 2013; Mateus-Pinheiro et al., 2013; Duman and Duman, 2014; Duman et al., 2016; Alves et al., 2017; Castrén and Antila, 2017; Levy et al., 2018; Levy et al., 2019). In fact, over the last years, several studies have evidenced the participation of neuroplasticity-related mechanisms as key players in the action of antidepressants, such as serotonin-reuptake inhibitors (Ampuero et al., 2010; Rubio et al., 2013; Mcavoy et al., 2015; Kitahara et al., 2016; Ampuero et al., 2019; Levy et al., 2019; Klöbl et al., 2022), electroconvulsive or hormonal therapies (Foy et al., 2010; Abbott et al., 2014; Catenaccio et al., 2016; Pirnia et al., 2016). Evidence has shown that these effects are associated with the upregulation of certain neurotrophins, like the mature brain-derived neurotrophic factor (mBDNF), which is known to be repressed by stress and is a critical mediator of antidepressant responses (Karege et al., 2002; Monteggia et al., 2004; Gonul et al., 2005; Duclot and Kabbaj, 2015; Ghosh et al., 2015; R. S; Duman et al., 2016; Castrén and Antila, 2017).

The administration of an aqueous extract of pomegranate (AE-PG) improves antidepressant-like effects of drugs of clinical use (Valdés-Sustaita et al., 2021). In a previous study, the administration of low doses of AE-PG combined with the antidepressant citalopram (CIT) synergized to produce an antidepressant-like effect in the forced swimming test (FST) in rats (Valdés-Sustaita et al., 2021), suggesting the participation of the serotoninergic system and estrogen receptors, specifically the beta receptors (ERβ), as a possible mechanism of action (Valdés-Sustaita et al., 2017; Valdés-Sustaita et al., 2021). It is noteworthy that ERβ has been considered an important target for antidepressant treatment and a mediator of the mechanism by which estrogen may influence neuronal plasticity (Liu et al., 2008; Zhao et al., 2011; Chhibber et al., 2017; Chidambaram et al., 2019). Nevertheless, neuroplasticity underlying these behavioral responses has not been explored at all.

The mBDNF is related to the dendrite spines, whose formation follows a fine-tuning process involving the sprouting of the membrane to generate filopodia, thin, stubby, and mushroom-head-type spines (Zhang and Benson, 2000; Kasai et al., 2003; Kasai et al., 2010; von Bohlen und Halbach, 2009). Interestingly, each of these morphological changes is associated with the formation of neuronal connections, information storage, and processing within the brain circuits that involves proteins such as synaptophysin (Valtorta et al., 2004; Zuo et al., 2005; O’Donnel et al., 2011; Hlushchenko et al., 2016; Runge et al., 2020). Thin-like spines, called small or immature spines due to their smaller head and narrow neck, have been considered learning spines capable of forming new memories during the synaptic plasticity process (Hayashi and Majewska, 2005; Bourne and Harris, 2007; Caroni et al., 2012). In contrast, mushroom or large spines, considered more stable, have the ability to form strong synaptic connections, capable of maintaining neuronal networks and long-term memory (Trachtenberg et al., 2002; Hayashi and Majewska, 2005; Bourne and Harris, 2007; Caroni et al., 2012). Regarding stubby spines, although having been classified as immature spines, some studies have suggested that this type of spines could be a form of active mushroom (Holtmaat et al., 2005; Caroni et al., 2012; Tønnesen et al., 2014). Finally, filopodia-shaped spines, which lack functional synapses, are considered important because they can still be found, mainly under specific conditions like induction of plasticity (Dailey and Smith, 1996; Ziv and Smith, 1996; Grutzendler et al., 2002; Zuo et al., 2005; Caroni et al., 2012). Considering that AE-PG exerts an antidepressant-like effect and potentiates the effects of clinical drugs such as CIT, in this study, their influence on neuroplastic changes in dendritic complexity, spines density, and neuroplasticity-related morphological changes was evaluated in the hippocampus using a selected combination. Also, mBDNF levels and expression of the synapse-related protein, synaptophysin, were measured in the hippocampus.

## Materials and methods

### Animals

Young adult (3-month-old) ovariectomized (OVX) female Wistar rats, weighing 210–260 g, were used in this study. Ovariectomy was performed by ventral approximation doing an incision on the skin and muscle of female rats under anesthesia with tribromoethanol (Estrada-Camarena et al., 2011). Oviducts and ovaries were located and after ligating the oviducts, ovaries were removed. Afterward, muscle and skin were sutured, rats received a dose of meloxicam to prevent surgery-related pain (Kim et al., 2023) and were left undisturbed for 3 weeks for recovery. All procedures related to animal care were in accordance with the Mexican official norm for animal care and handling (NOM-062-ZOO-1999) and approved by the Institutional Ethics Committee of the *Instituto Nacional de Psiquiatria “Ramón de la Fuente Muñiz"* (CEI-200/2015). The experimental work was developed in Mexico City (2,240 m above sea level) from March to April (Atmospheric pressure = 1,022.6 Pa; Servicio Meteorológico Nacional, 2023 Mexico City). During the whole experimental process, animals were maintained in standard laboratory cages (5 animals per cage) under a 12-h light/12-h dark cycle (starting the light cycle at 22:00 and ending at 10:00 h) at a temperature of 23 ± 1°C, and with free access to food and water. The same person manipulated the animals daily for approximately 15 min during the treatment administration.

### Experimental design

**FIGURE 1:**
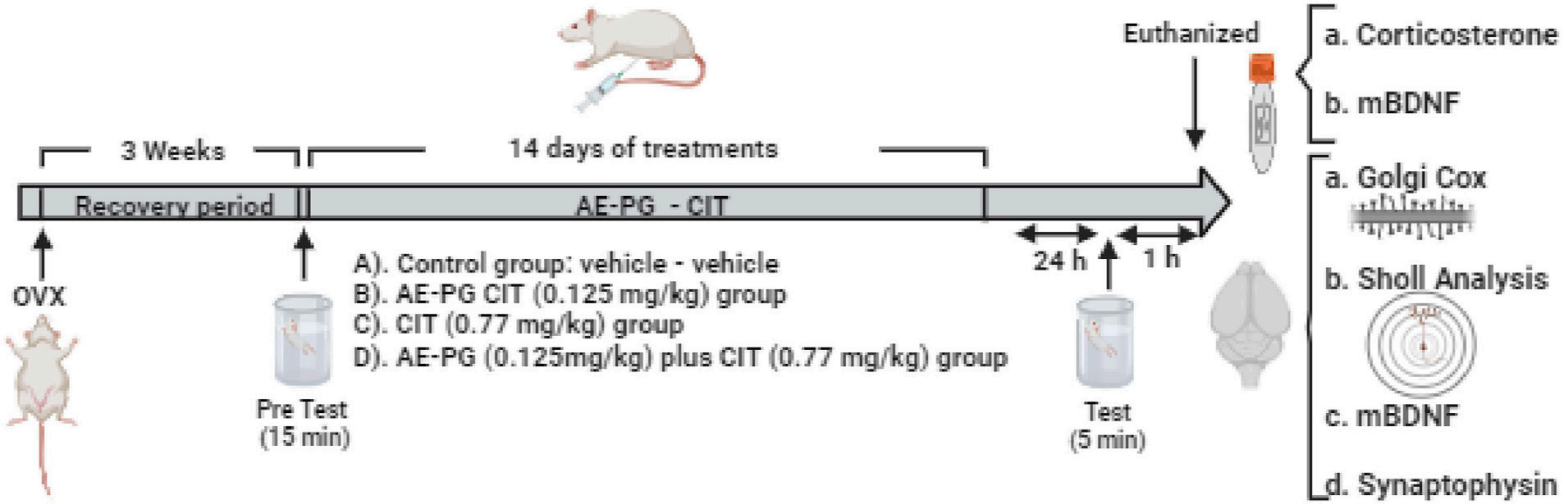
Schematic representation of the experimental design used to evaluate the effects of the combination of AE-PG plus CIT at sub-optimal doses on the depressive-like behavior induced by the forced swimming test (FST), plasma corticosterone and mBDNF, synaptophysin protein expression and mBDNF in hippocampus, and dendritic complexity, density, and morphology of dendritic spines in the dentate gyrus of the ovariectomized rat. Created in BioRender.com.

As shown in Figure 1, 3 weeks after the OVX, the animals were exposed to an acute stress session (15 min) induced by a forced swimming test and, subsequently, animals were randomly assigned to the respective treatment. Based on Valdés-Sustaita et al. (2017), the DE_30_ for CIT and AE-PG was calculated and dose-response curves were constructed with independent groups of rats (n = 8–10 per group) to select a non-effective dose for the combinations on FST. Doses for CIT (0.77, 3.06, and 12.24 mg/kg) and AE-PG (0.125, 0.50, and 2.0 mg/kg) were combined in a 1:1 proportion, and only the combination of drugs that alone did not produce behavioral effects on FST were processed for neuroplastic analysis. In all cases, AE-PG, CIT, their combination, and/or the saline solution were administered chronically for 14 days (once a day) from 9 to 10 a.m. (Valdes-Sustaita et al., 2017; Valdes-Sustaita et al., 2021). One day after completing the administration, the animals were subjected to the open field test to discard a motor alteration that could interfere with the response of animals in the FST (Estrada-Camarena et al., 2002); 30 min later, a second forced swimming test session (5 min) was performed to evaluate ability to cope with a stressful situation.

Hence, the animals analyzed for the neuroplastic effect were groped in: A) Control group (saline solution [s.s.], 0.9% NaCl) plus distilled water, n = 15), B) AE-PG group at a sub-threshold dose (s.s Plus AE-PG 0.125 mg/kg, n = 12), C) CIT group at a sub-threshold dose (CIT 0.77 mg/kg plus vehicle of AE-PG, n = 15), and D) combination of a sub-threshold dose of AE-PG plus CIT (0.125 mg/kg and 0.77 mg/kg, respectively; n = 12). At the end of the behavioral test, animals were euthanized by decapitation and their brain collected and prepared to analyze the formation and maturation of dendrite spines on granule cell dendrites and changes in the dendritic complexity (n = 4–5). Blood was collected to evaluate corticosterone and mBDNF (n = 4–6), and the hippocampus was dissected, divided into right and left hemisphere, and frozen at −70°C until evaluating mBDNF levels and synaptophysin protein expression (n = 4–6).

### Behavior test

#### Open-field test

To discard a possible influence of drug treatments on locomotor activity, the effect of the AE-PG and CIT drugs, alone or in combination, was tested in the open-field test for 5 min. The test consisted in placing the animals individually on an opaque-Plexiglas box (40 × 50 × 30 cm^3^) with the floor divided into 12 equal squares (12 × 12 cm^2^) on which the animals could walk freely. The number of times the animal crossed the squares during a 5-min session were registered and analyzed (Estrada-Camarena et al., 2002).

### Forced swimming test (FST)

The FST consisted in two swimming sessions separated by 14 days (Detke et al., 1997; Estrada-Camarena et al., 2008; Vega-Rivera et al., 2013; Vega-Rivera et al., 2015). On the first session (pre-test), rats were forced to swim for 15 min to induce a state of stress in them. Fourteen days after, on a second session (test), the animals were re-exposed to the forced swimming for 5 min to evaluate the effects of treatment. In both sessions, the animals were placed in Plexiglass cylinders (20 cm in diameter and 46 cm tall, filled with a 30-cm water layer at 23°C ± 2°C; after each swimming session, animals were dried and then placed in their home cages, changing the water of the cylinder to avoid any influence on the next rat (Bogdanova et al., 2013). Considering that the rodents were less active and stressed during the dark phase, both sessions were carried out at the beginning of the dark cycle (10:00 to 12:00 h). The 5-min test was video recorded, and three behaviors were scored by an observer unaware of treatments in 5-s intervals until completing 5 min: 1) Immobility, which was defined as minimal movements exerted by the animal to keep its head above the water and float; 2) swimming, defined as movement of forepaws to displace the body along the swimming cylinder, and 3) climbing, defined as vigorous movements with forepaws in and out of the water along the cylinder walls (Porsolt et al., 1977; Lino-De-Oliveira et al., 2005). FST sessions were carried out in a separate room used only for the scoring of the behavior. Then animals remained in a “waiting room” for holding prior to the behavioral test and, then, were moved to a next room to perform the behavioral test. Also, to ensure constant control of the environment and to minimize interference that may modify experimental results (Bogdanova et al., 2013) only the experimenter had access to the behavioral room.

### Drugs and chemicals

Citalopram (Sigma-Aldrich, Mexico) was dissolved in physiological saline solution (0.9% NaCl), AE-PG (Nutracitrus SL, Elche, Alicante, Spain) was freshly prepared using distilled water. CIT and AE-PG were administered intraperitoneally (i.p.) in a volume of 4 mL/kg body weight of the animals.

### Determination of BDNF and corticosterone

Corticosterone (CORT) was determined in plasma samples, and mBDNF levels were quantified from plasma and hippocampal tissue. Blood samples were obtained by decapitation and kept on ice until centrifugation (4,000 rpm at 4°C for 15 min) to allow plasma extraction. Plasma was kept at −80°C until analyzed with a commercial ELISA corticosterone (Enzo Life Sciences, Farmingdale, NY, United States) and BDNF immunoassay kit (EMD Merck-Millipore Corporation, Darmstadt, Germany) according to manufacturer’s instructions. The right hemisphere containing hippocampal tissue was homogenized in lysis buffer (RIPA Lysis Buffer System: sc-24948S) with an ultrasonic apparatus. The homogenates were centrifuged at 14,000 rpm for 15 min at 4°C, and the supernatants were collected to determine the mBDNF with a commercial ELISA immunoassay kit (EMD Merck-Millipore) and total protein concentration using Bradford reagents and bovine serum albumin (BSA) as the standard. Microplates were read at 405, 450, and 595 nm in an ELISA reader (BioTek Instruments, Winooski, VT, United States).

### Western blot analyses of synaptophysin protein

For total protein extraction, hippocampal tissue was homogenized with 300 µL of RIPA lysis buffer containing protease inhibitors (Santa Cruz Biotechnology, Dallas, TX, United States). A total of 60 μg of proteins was separated by electrophoresis on a 12% SDS-PAGE gel and transferred to a nitrocellulose membrane. Membranes were blocked and blots were tested using primary antibodies including synaptophysin (Abcam, ab14692; 1:500) and Anti-GAPDH (FNab 03,343, Fine Test; 1:5,000). All antibodies were incubated overnight, the next day the membrane was washed with TBST; the Goat-anti-mouse HRP secondary antibody (Advansta) was incubated for 1 h at room temperature. Membranes were washed again; images were obtained using ChemiDoc XRS+ (Bio-Rad, Hercules, Ca, United States). The relative density of the specific bands was quantified using Image Lab 6.1 software (Bio-Rad).

### Brain golgi staining

To evaluate neuroplasticity reflected by density and type of dendritic spines, the animals were euthanized by decapitation (n = 4–5), their brains were removed and the mid-brain of each animal was processed for Golgi-Cox with the FD Rapid Golgi Stain TM kit according to manufacturer’s instructions of (FD NeuroTechnologies, Columbia, MD, United States) (Das et al., 2013; Risher et al., 2014; Zaqout and Kaindl, 2016; Du, 2019). The tissue was sectioned using a microtome (Leica SM 2010R; Leica Biosystems, Deer Park, IL, United States) to obtain coronal sections of 200 μm of thickness from the entire hippocampus (AP: −2.3 to −4.5 from bregma) (Paxinos and Watson, 2007). The tissue sections were mounted onto gelatin-coated glass slides to develop thereafter the Golgi-Cox impregnation with NH_4_OH and, subsequently, dehydrated through a series of graded ethanol washes and cleared in NeoClear™ (Merck, United States) to be covered with mounting medium (Neumount; Merck, United States) and left in the dark. From each animal, 10 representative neurons, located within the granular layer of the hippocampal dentate gyrus, were selected at random using a Leica (Leica Microsystems, Inc., Buffalo Grove, IL, United States) microscope at 100X. Density and the type of dendritic spines were analyzed in 20 μm of length of the secondary dendrite of each neuron using the Reconstructor^®^ software (Fiala, 2005; Risher et al., 2014). The classification of type of dendritic spine was based on a critical formula to determine length and length-to-width ratio (LWR) divided by the width value of an individual spine (Risher et al., 2014).

### Sholl analysis

To determine whether AE-PG and CIT at sub-threshold doses were able to induce changes in the dendritic complexity of Golgi-impregnated neurons of the hippocampal dentate gyrus, the branching pattern and length of the dendritic trees of seven different Golgi-impregnated neurons per animal were evaluated by the Sholl’s concentric circles technique as previously reported (Vega-Rivera et al., 2013). For this analysis, we only considered neurons with vertical-oriented dendrites with their arborizations extending through the granular cell layer and reaching the molecular layer (ML) and being relatively isolated from neighboring impregnated cells (Vega-Rivera et al., 2015). These neurons were selected using a light microscope (Leica DMLS, Germany) coupled to a digital DM50 camera. The FijiJ software (Image processing and Analysis in Java; NIH Bethesda, MA, United States) was used to convert the branches observed in three dimensions to a two-dimensional image and calculate dendritic complexity, reflected by dendritic length and the number of intersections (Vega-Rivera et al., 2015).

### Statistical analysis

Results are shown as the mean ± standard error of the mean (S.E.M). Comparisons among groups were done using the Sigma Plot 12.0 software (Systat Software Inc., Chicago, IL, United States). The behavioral analysis, the hormone levels, protein expression, dendritic complexity, density, and category of dendritic spines with Golgi-Cox impregnation were analyzed with one-way analysis of variance (ANOVA) followed by Holm-Sidak *post hoc* test. Data from the Sholl technique were analyzed by a two-way ANOVA followed by Tukey´s *post hoc* test, considering the treatment and the number of dendritic branches as testing factors. In all cases, the statistical level of significance was set at *p* < 0.05.

## Results

### Dose response curves of DE_30_ of AE-PG and CIT on FST

As depicted on Table 1, from the DE_30_ of AE-PG only the dose of 2 mg/kg significantly reduced the immobility behavior on FST (F:_3,34_ = 9.03, *p* < 0.001) with a concomitant increase in climbing behavior (F:_3,34_ = 5.43, *p* = 0.04) compared against the control group, without significant changes on swimming at any dose. CIT reduced the immobility behavior at the 12.04 mg/kg dose (F:_3,41_ = 9.39, *p* < 0.001), increasing swimming (F:_3,41_ = 7.80, *p* < 0.001) and climbing (F:_3,41_ = 4.40, *p* = 0.01) in comparison to the control group (Table 1).

**TABLE 1:** Effect of Citalopram, AE-PG alone or in combination on immobility behavior and locomotor activity.

Treatment (mg/kg)	Immobility	Swimming	Climbing	Number of squares crossed in 5-min
CONTROL	43.60 ± 1.82	11.26 ± 1.11	5.13 ± 1.28	43.25 ± 8.08
CIT 0.77	41.66 ± 1.58	12.33 ± 1.05	6.00 ± 1.09	39.20 ± 6.43
CIT 3.06	38.10 ± 2.74	17.40 ± 2.40*	4.50 ± 0.88	ND
CIT 12.04	24.00 ± 4.13**	23.60 ± 3.57**	12.4 ± 2.42*	34.60 ± 3.76
*One-Way Anova test*	*F:*_*3,41*_ *= 9.39, p < 0.001*	*F:*_*3,41*_ *= 7.80, p < 0.001*	*F:*_*3.41*_ *= 4.40, p = 0.01*	*F:*_*2,20*_ *= 0.36, ns*
CONTROL	43.09 ± 1.59	13.09 ± 0.95	3.81 ± 0.93	30.44 ± 5.18
AE-PG 0.125	39.75 ± 2.88	13.66 ± 2.16	6.58 ± 1.50	47.00 ± 6.80
AE-PG 0.50	34.44 ± 2.51*	17.00 ± 2.42	8.10 ± 1.75	ND
AE-PG 2.0	21.40 ± 3.80**	21.60 ± 4.20	17.0 ± 5.52*	34.60 ± 3.76
*One-Way Anova test*	*F:*_*3, 34*_ *= 9.03, p < 0.001*	*F:*_*3,34*_ *= 2.21,ns*	*F:*_*3,34*_ *= 5.43, p = 0.04*	*F:*_*2.21*_ *= 2.59, ns*
CONTROL	41.25 ± 2.75	12.25 ± 10.6	6.50 ± 1.96	40.06 ± 2.84
CIT (0.77)+ (AE-PG 0.125)	26.86 ± 1.88**	24.41 ± 1.15*	8.75 ± 2.02	22.41 ± 5.18
CIT (3.06)+ AE-PG (0.50)	33.30 ± 1.62*	21.50 ± 1.49*	5.20 ± 0.85	ND
CIT (12.04)+ AE-PG (2.0)	22.40 ± 5.16**	25.20 ± 3.18*	12.40 ± 3.88	38.60 ± 5.38
*One-Way Anova test*	*F:*_*3,31*_ *= 9.11, p < 0.001*	*F:*_*3,31*_ *= 13.5,p < 0.001*	*F:3,31 = 1.81, ns*	*F:*_*2,17*_ *= 0.07, ns*

Data are presented as mean ± error standard median of number of immobilities scored in a 5-min forced swimming test and of the number of squares crossed in a 5-min. CIT, citalopram; AE-PG, Aqueous extract of *Punica granatum*. *p < 0.05; **p < 0.005 Holm-Sidack *post hoc* test. One Way ANOVA values are presented in italic.

Next, three combinations of AE-PG and CIT (Table 1; 0.125 + 0.77, 0.5 + 3.06 and 2.0 + 12.04 mg/kg, respectively) were tested. All combinations were effective in reducing the immobility behavior (F:_3,31_ = 9.11, *p* < 0.001) and increasing the swimming behavior (F:_3,31_ = 13.5, *p* < 0.001) as compared against the control group. No changes were observed in climbing behavior with any combination, and no differences were observed among them.

Finally, no changes in general activity were observed with any treatment either alone or in combination (Table 1).

### Combining AE-PG and CIT improves coping with inescapable stress in the forced-swim test

**FIGURE 2:**
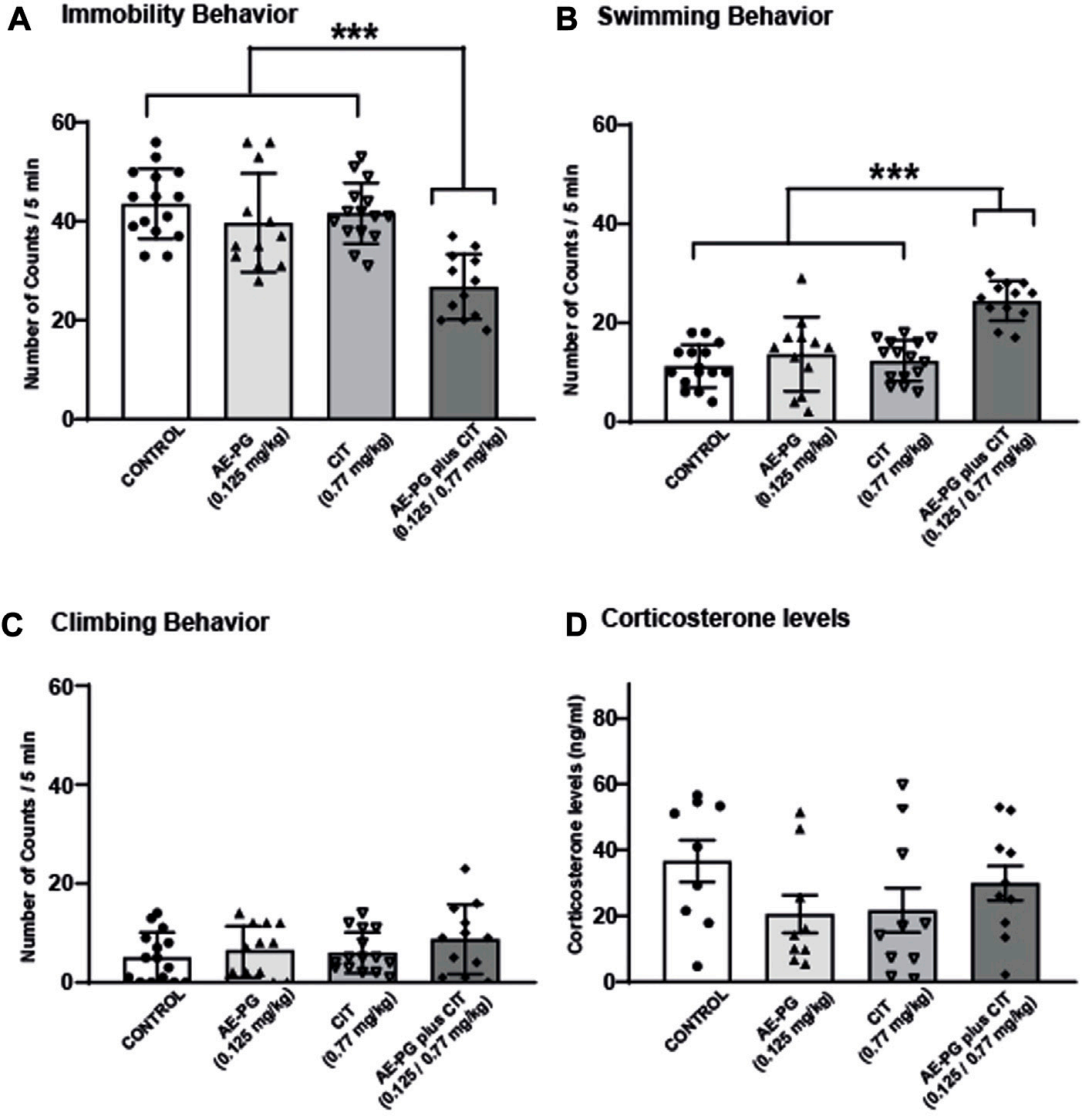
The combination of AE-PG plus CIT reduces the immobility behavior induced by FST. Rats were treated with AE-PG (0.125 mg/kg, orally administered), CIT (0.77 mg/kg; i.p.), or with the combination of AE-PG (0.125 mg/kg) plus CIT (0.77 mg/kg) for 14 days Data are expressed as the mean number of counts ± SEM of immobility **(A)**, swimming **(B)**, and climbing **(C)** in a 5 min test period of n = 12–14 per group. Panel **(D)** show the effect of treatments on corticosterone levels of n = 4-5 animals per group. Two Way ANOVA test followed by Tukey *post hoc* test, ***p ≤ 0.001.

In a second experiment, the effects of AE-PG (0.125 mg/kg) and CIT (0.77 mg/kg) either alone or in combination were tested on the animals’ ability to cope with an acute stressful situation in the FST (Figures 2A–C). Confirming our previous data, AE-PG (0.125 mg/kg) and CIT (0.77 mg/kg) alone did not modify the immobility behavior (Figure 2A, non-significant) nor the swimming behavior (Figure 2B, non-significant) when compared to the control group. Whereas the combination of AE-PG plus CIT (0.125 mg/kg and 0.77 mg/kg) significantly (*p* < 0.001) decreased the immobility behavior in the FST, increasing the swimming behavior (*p* < 0.001). Neither AE-PG, CIT, nor the combination AE-PG plus CIT modified the climbing behavior (Figure 2C, non-significant). One-way ANOVA analysis yielded the following values: for immobility F_3,50_ = 13.075, *p* < 0.001; swimming F_3,50_ = 18.133, *p* < 0.001; and for climbing F_3,50_ = 1.085, non-significant.

Effect of the combination of AE-PG plus CIT on dendritic complexity of granule cells in the dentate gyrus: Sholl analysis.

**FIGURE 3:**
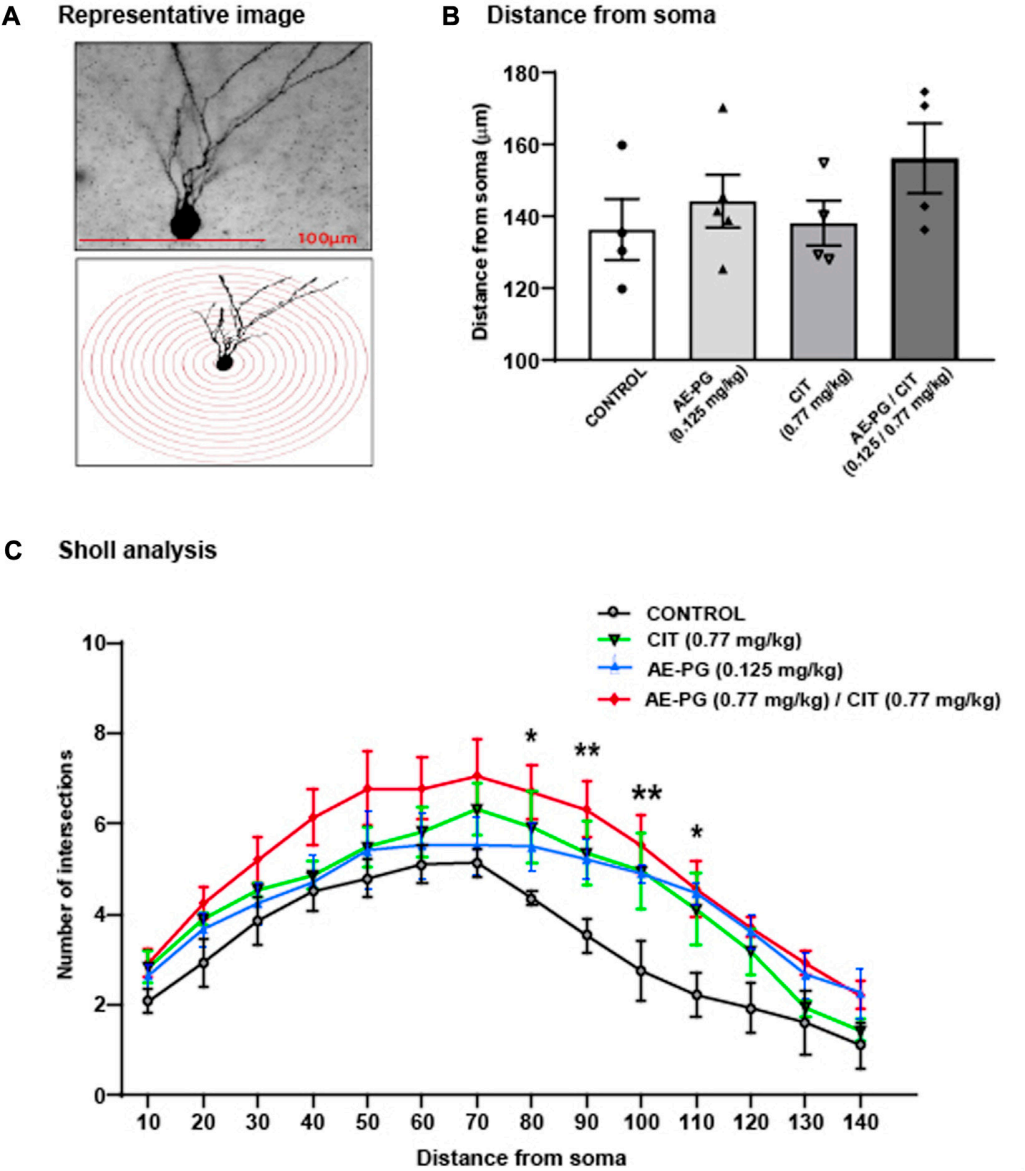
Effect of the combination of AE-PG plus CIT on dendritic complexity of granule cells in the dentate gyrus: Sholl Analysis **(A)** Representative photomicrographs of Golgi-impregnated neurons and their respective illustrative drawing of concentric Sholl circles superimposed on the granular neuron to determine the number of intersections **(B)** The dendrite complexity of Golgi-impregnated neurons is represented by the mean total number of intersections ± SEM concerning the soma distance **(C)** Full dendritic length of Golgi-impregnated neurons in the dentate gyrus of the hippocampus. Two Way ANOVA test followed by a Holm Sidak *post hoc* test. The bracket shows the differences between the group that received the combination AE-PG plus CIT at doses sub-optimal and the rest of the groups: *p ≤ 0.05 vs control; n = 28 (four animals per treatment, seven neurons per animal).

The analysis of the dendritic complexity of Golgi-impregnated neurons in rats treated with AE-PG, CIT, or their combination (Figures 3A,B) revealed the main effects of the treatment (F_3,237_ = 26.325, *p* < 0.001). Here, the combination AE-PG plus CIT showed higher effects on the dendritic complexity than in the control group (*p* = 0.001). Similar effects were seen after the comparison with AE-PG (*p* < 0.001) or CIT (*p* < 0.001).

In addition, the *post hoc* analysis of the number of intersections from soma along the dendrite showed a relevant effect of the distance (F_3,237_ = 28.097, *p* < 0.001) (Figures 3A,B). The combination AE-PG plus CIT increased the number of dendritic branches from 80 µm (*p* = 0.014) to 110 µm (90 μm, *p* = 0.002; 100 μm, *p* = 0.002; and 110 μm, *p* = 0.012) compared to the control group. Two-way ANOVA revealed: interaction of treatment × distance was not significant, F_3,237_ = 0.560, *p* = 0.983. Concerning the length of the longest dendrites of hippocampal neurons, we observed no statistically significant difference among the groups (Figure 3C, *p* = 0.355).

### Effects of the combination of AE-PG plus CIT on density and maturation of dendritic spines

**FIGURE 4:**
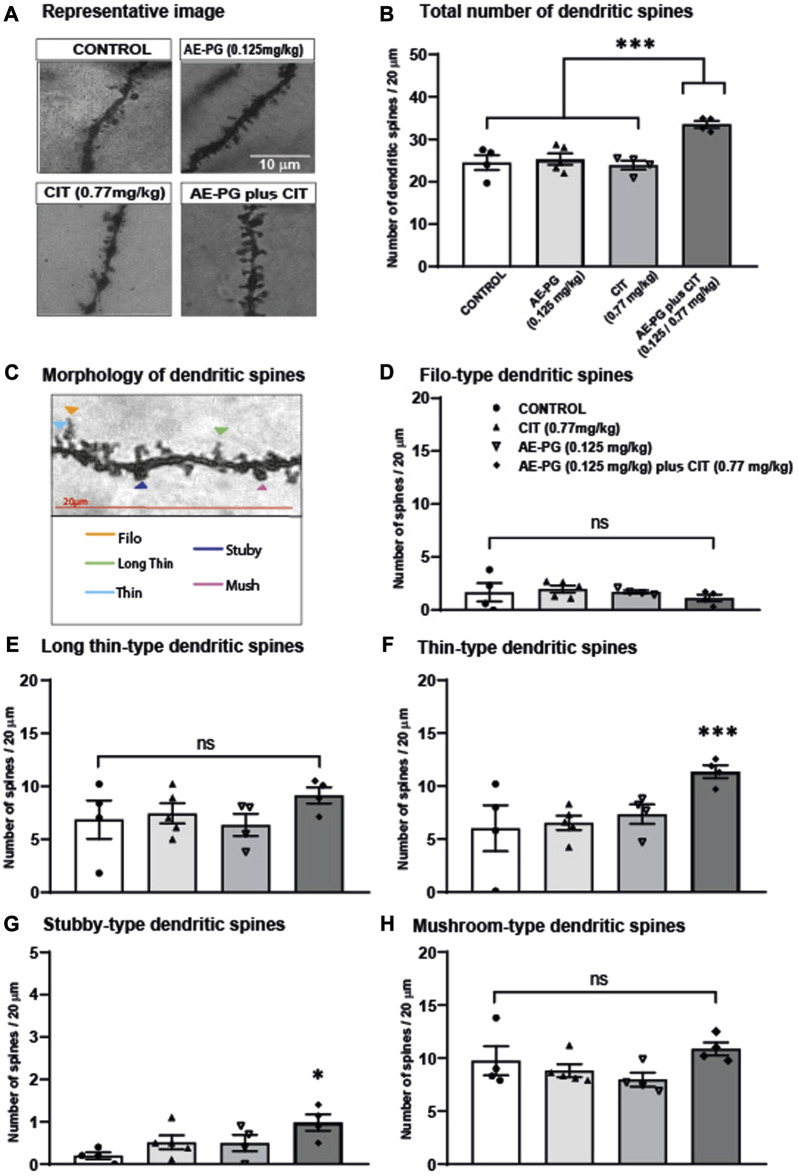
Effects of the combination of AE-PG plus CIT on density and maturation of dendritic spines **(A)** Representative photomicrographs of dendritic spines along secondary dendrite of neurons of different treatments. Scale bar = 10 µm **(B)** The number of spines was quantified along 20 μm of the dendrite of neurons from control, AE-PG, CIT, and the combination AE-PG plus CIT **(C)** Representative photomicrographs of five types of dendritic spines based on their head and neck morphology (filo: long and thin protrusions without a bulbous head; long thin; thin: smaller head and a narrow neck; stubby: large bulbous head and a short wide neck; mushroom: Characterized by a large bulbous head and a short narrow neck) **(D–H)** Effects of combination AE-PG plus CIT at doses sub-optimal on filo **(D)**, long thin **(E)**, thin **(F)**, stubby **(G)**, and mushroom **(H)** spines number along 20 μm of the dendrite of neurons from control, AE-PG, CIT, and the combination AE-PG plus CIT. The data represents the mean of the total number of dendritic spines ±SEM. One Way ANOVA test followed by Tukey, *p ≤ 0.05; ***p ≤ 0.001.

In addition to the analysis of the dendritic complexity, the structure of dendritic spines along dendrites was analyzed in granule cells (Figure 4). Quantification of the dendritic spines along 20 μm of the dendrite revealed that the combination of AE-PG plus CIT significantly increased (F_3,16_ = 11.508, *p* < 0.001; Figures 4A,B) the number of dendritic spines compared with the control group (*p* = 0.002), AE-PG (*p* = 0.003), or CIT (*p* = 0.001). The analysis of the morphology of dendritic spines revealed differences in some stages of their development (Figures 4C–F). Following the course of the generation of dendritic spines, the analysis of filopodium and long-thin spine categories did not show differences among the groups (F_3,16_ = 0.560, non-significant; F_3,16_ = 0.99, non-significant, respectively). However, the analysis of the categories thin (Figure 4E) and stubby (Figure 4F) showed an increased number in both categories in rats treated with the combination of AE-PG plus CIT compared to the control group (*p* = 0.041, *p* = 0.028, respectively). The other groups did not show differences (F_3,16_ = 3.90, *p* = 0.034; F_3,16=_3.60, *p* = 0.43, respectively). Finally, the treatments did not affect the latest stage of dendritic spines development identified through the mushroom morphology (F_3,16_ = 2 .057, *p* = 0.156; Figures 4A,B).

The combination of AE-PG plus CIT increases mBDNF concentrations and synaptophysin protein expression in the hippocampus but not in CORT or mBDNF in plasma.

**FIGURE 5:**
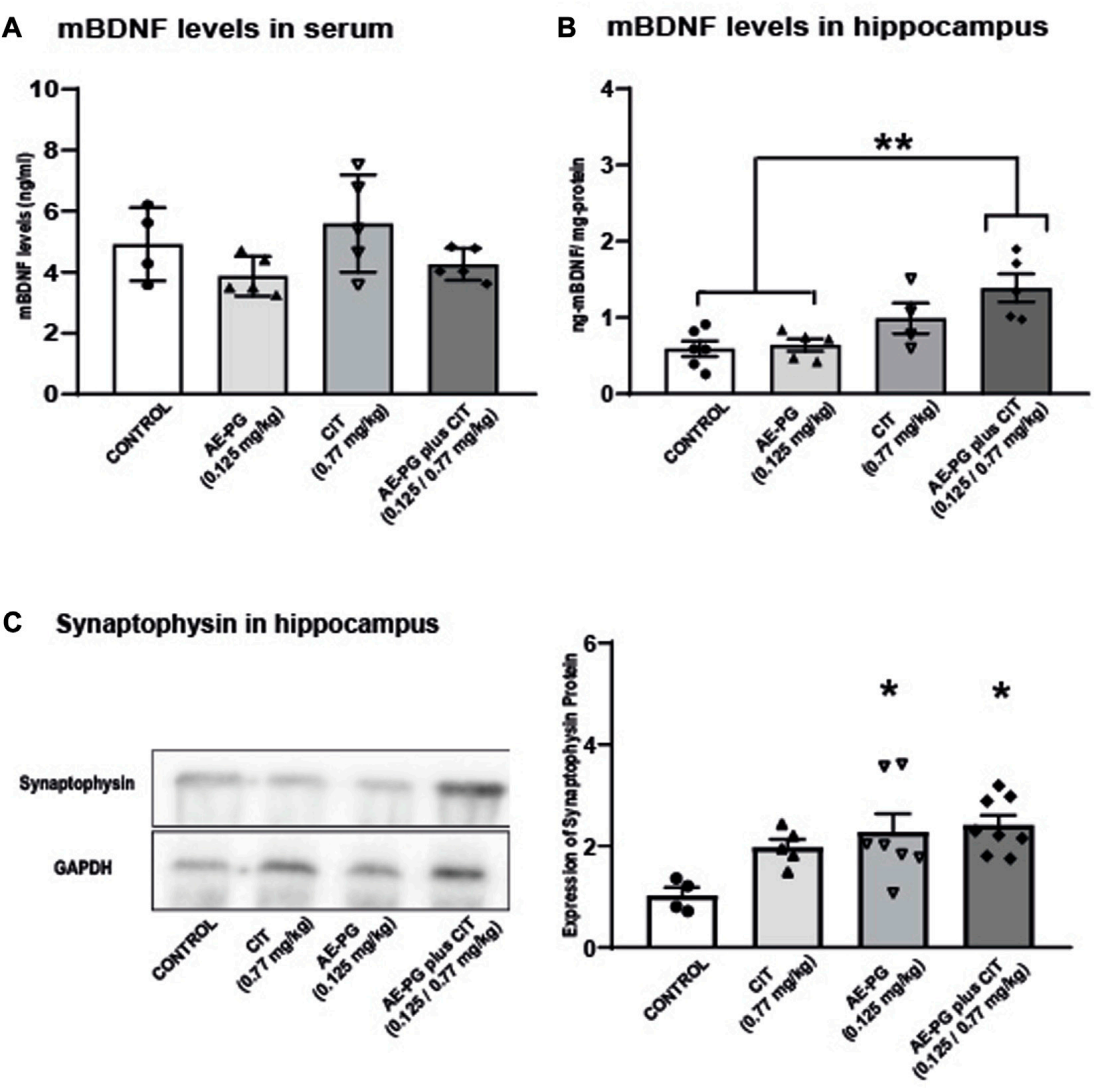
Effects of the combination of AE-PG plus CIT on mBDNF on plasma **(A)** and hippocampus **(B)** and synaptophysin protein expression on hippocampal tissue **(C)** of ovariectomized rats. Representative immunoblot of synaptophysin protein expression. Data are expressed as the mean ±SEM of n = 4-6 animals per group. One Way ANOVA test followed by Holm-Sidack, *p < −0.05.

Quantification of CORT as an index of activation of the hypothalamic-pituitary-adrenal (HPA) axis revealed that chronic administration of AE-PG, CIT, and the combination AE-PG plus CIT at non-effective doses were not statistically significant different when compared among groups (Figure 2D; F_3,34_ = 1.518, *p* = 0.228). Like corticosterone levels in serum, mBDNF levels were not significantly different among the different groups (Figure 5A; F_3,18_ = 2.478, *p* = 0.101). However, differences among the groups were observed in hippocampal tissue (F_3,20_ = 7.102, *p* = 0.003; Figure 5B). Here the combination of AE-PG plus CIT significantly increased the mBDNF concentrations compared with the control group (*p* = 0.004) and AE-PG (*p* = 0.009), but not with CIT (*p* = 0.278).

Finally, the expression of synaptophysin, a protein index of synaptogenesis, was analyzed in the hippocampus. As seen, AE-PG alone (*p* < 0.03) or its combination with CIT (*p* < 0.01) increased the expression of synaptophysin in samples of the hippocampus (F:_3,20_ = 4.48, *p* = 0.01; Figure 5C) when compared against the control group, without differences between them.

## Discussion

Our present results evidence that a combination of subthreshold doses of AE-PG plus CIT produced antidepressant-like effects by diminishing the immobility behavior associated to an increase in the complexity of the dendrites of the granule neurons at the granular cell layer in the rat hippocampus. Particularly, a significant increase was observed in the number of dendritic spines of thin and stubby categories associated to an increase in hippocampal mBDNF concentrations and synaptophysin protein expression.

### Antidepressant-like effects of a combination of AE-PG plus CIT

Previous studies revealed that AE-PG induces antidepressant-like effects in the FST in OVX rodents (Mori-Okamoto et al., 2004; Valdés-Sustaita et al., 2017) and synergizes the effects of non-pharmacological compounds, such as *Citrus limon* (Riaz and Khan, 2017), or antidepressant clinical drugs like CIT (Valdés-Sustaita et al., 2017). In the present work, we confirmed that a combination of non-effective doses of AE-PG plus CIT produced significant antidepressant-like effects not observed in the administration of AE-PG or CIT alone. This effect is likely produced through the participation of the serotonergic system because an increase in swimming behavior was found in animals treated with the combination of AE-PG plus CIT. To this respect, preliminary reports demonstrated that antidepressant-like effects of AE-PG were associated with the serotonergic system and β-estrogen receptors in the FST (Valdés-Sustaita et al., 2017; Valdés-Sustaita et al., 2021). Notably, ERβ agonists and E2 promoted the same behavioral profile that serotonergic compounds in the FST (Detke et al., 1995; Estrada-Camarena et al., 2003; Valdés-Sustaita et al., 2021). Further, estradiol modulated the serotonergic system and facilitated the antidepressant action of fluoxetine (FLX) in the same tests (Estrada-Camarena et al., 2006a; Estrada-Camarena et al., 2006b). Thus, our results suggest that antidepressant-like effects of AE-PG plus CIT involve the serotonergic system in the FST. Also, two active compounds found in the used AE-PG are ellagic acid and punicalagin, which induce a behavioral profile like that induced by AE-PG, i.e., decrease of immobility plus increase of swimming (Cervantes-Anaya et al., 2022). Both compounds are ellagitannins with estrogenic activity, and at least ellagic acid is also active in the serotonergic system (Dhingra and Chhillar, 2012). Specific experiments are warranted to prove this assumption.

## The combination of AE-PG plus CIT modifies dendritic complexity of the granular neurons in the granular cell layer

The present study showed that the antidepressant-like effect produced by the combination of suboptimal dose of AE-P plus CIT occurred with an increased dendritic complexity of Golgi-impregnated neurons of the granule cell layer in the hippocampus. In this sense, several studies evidenced that therapeutic effects of antidepressant drugs are associated with dendrite restructuration and maturation (Norrholm and Ouimet, 2001; Malberg and Duman, 2003; Chen et al., 2006; Plümpe et al., 2006; Wang et al., 2008; Guirado et al., 2012; Vega-Rivera et al., 2015). For instance, an effective dose of FLX produces antidepressant-like actions concomitantly increasing the dendritic tree complexity in newborn neurons in the hippocampus after at least 21 days of treatment (Wang et al., 2008; Guirado et al., 2012). Previously, it was shown that E2 synergizes with FLX inducing an antidepressant-like action associated with increased dendritic complexity (Vega-Rivera et al., 2015). Thus, it is possible that the phytoestrogens present in the AE-PG could contribute to stimulate actions on the dendritic tree complexity and facilitate the action of CIT in 14 days. Supporting this, the antidepressant-like action of AE-PG is blocked by non-selective estrogen receptors (tamoxifen) and the ERβ-antagonist (PHTPP) (Valdés-Sustaita et al., 2017; Valdés-Sustaita et al., 2021).

The increased dendritic complexity found in granule neurons in rodents treated with the combination of suboptimal doses of AE-PG plus CIT may occur, directly or indirectly, through the serotonergic system (Rojas et al., 2017), which is also modulated by the activation of ERβ (Rybaczyk et al., 2005). The activation of ERβ increases the hippocampal dendritic complexity through the regulation of signaling pathways involved in the cytoskeleton rearrangement (Srivastava et al., 2010; Rojas et al., 2017). The behavioral profile observed in the present study (increase of swimming behavior) induced by the combination of AE-PG and CIT suggests the involvement of the serotonergic system in modulating the dendritic complexity and the antidepressant-like action in OVX rats. Specific experiments are necessary to confirm this hypothesis.

The combination of AE-PG plus CIT modifies the density and the morphology of dendritic spines in granular neurons in the granular cell layer.

Results showed that antidepressant-like effects produced by the combination of suboptimal doses of AE-PG plus CIT are associated with an increased density of dendritic spines in the hippocampus. Evidence has shown that an increase in spines density is associated with restructuration of synaptic connectivity and improves maladaptive behavior and learning tasks (Moser et al., 1994; Moser et al., 1997; Bruel-Jungerman et al., 2005; Kozorovitskiy et al., 2005; von Bohlen und Halbach, 2009; Chidambaram et al., 2019). Hence, the increase in the spine’s density induced by the combination of AE-PG plus CIT, at suboptimal doses, could be forming more synaptic contacts and, therefore, improving a neuronal connection permitting a better behavioral response in rats exposed to a stressful situation, such as the one elicited by the FST. Furthermore, the reestablishment of the dendritic spine number and shape are considered the basis for the restoration of behavioral homeostasis induced by antidepressants (Norrholm and Ouimet, 2001; Hajszan et al., 2005; Mcavoy et al., 2015), estrogens, and phytoestrogens like resveratrol (Gould et al., 1990; Woolley and McEwen, 1994; Murphy and Segal, 1996; Li et al., 2021).

The combination of AE-PG plus CIT influences the morphology of dendritic spines in granular neurons in the granular cell layer.

It is suggested that an increase in interneural connectivity depends on the shape, not the increase in the number of spines. Evidence showed that morphological changes of spines are associated to maturation and stabilization of synapsis (Peters and Kaiserman-Abramof, 1970; Kasai et al., 2003; Tashiro and Yuste, 2003; Nimchinsky et al., 2004; Duman and Duman, 2014; Hlushchenko et al., 2016; Chidambaram et al., 2019; Runge et al., 2020), and that remodeling of dendritic spines is related to fast behavioral effects of antidepressant treatments (Hajszan et al., 2005; Bessa et al., 2009; O’Leary et al., 2009; Duman and Duman, 2014; Moda-Sava et al., 2019). In relation to this, behavioral effects of antidepressant drugs correlate with dendritic spines known as mushroom-like spines, which have been associated to stronger and longer lasting synaptic connections (Ampuero et al., 2010; Wang et al., 2013). From this, reduction of the immobility behavior observed in the AE-PG and CIT combination might be associated with the increase of mushroom spines. Contrary to this, the present results showed that the combination of suboptimal doses of AE-PG plus CIT increases the number of thin or stubby-like dendrites in rats that showed low immobility behavior. Other reports indicate similar findings. For example, the anxiety/depressive-like phenotype induced by chronic exposure to corticosterone (35 days) has been associated with a reduction in thin and stubby spines density, but not mushroom; accordingly, this reduction was reversed by long-term treatment with FLX (Wang et al., 2013). Also, studies in hippocampal neuronal slices of 12-week-old male rats showed that, after treatment with E2, the density of thin spines increased, but not that of mushroom and stubby spines (Mukai et al., 2007).

The fact that the combination of AE-PG and CIT increases dendritic spines and dendritic tree complexity and favors the reduction of immobility behavior suggests that this treatment contributes to develop strategies to cope with stressful situations. In this sense, growing evidence indicates why thin-like spines are also called “learning spines” for their information-acquiring ability during the synaptic plasticity process (Trachtenberg et al., 2002; Kasai et al., 2003; Matsuzaki et al., 2004; Bourne and Harris, 2007). After stimulation, the immature dendritic spines can transit to stable mushroom-like dendritic spines, achieving solid appraisal of the situation and rapid acquisition of new memory (Matsuzaki et al., 2004). Here, it is possible that the antidepressant-like effect of the AE-PG plus CIT combination involves the presence of more thin dendritic spines, which would receive the information given by the exposure to force swimming test test and induce their maturation. However, this assumption needs to be analyzed in additional studies.

Different studies have shown that regulation of morphological changes in spines is associated with the maturation and stabilization of synapsis in the central nervous system and could be mBDNF-dependent (von Bohlen and Halbach, 2009; von Bohlen und Halbach and von Bohlen und Halbach, 2018; Zhang and Benson, 2000; Kasai et al., 2003; Kasai et al., 2010). It has been demonstrated that mBDNF has a critical role in the behavioral and cellular efficacy of different antidepressant treatments, including selective serotonin reuptake inhibitors, such as FLX (Li et al., 2017; Saarelainen et al., 2003; Sairanen et al., 2005; Shirayama et al., 2002; Gonul et al., 2005; Duclot and Kabbaj, 2015; Ghosh et al., 2015; Duman et al., 2016; Castrén and Antila, 2017). Further, expression of mBDNF in structures of the limbic system, such as the hippocampus, is upregulated after a chronic treatment with antidepressants (Nibuya et al., 1995; Nibuya et al., 1996). Several findings indicate that serum BDNF is a biomarker of antidepressant efficacy (Levada et al., 2016). In this sense, studies have suggested that blood and plasma mBDNF levels reflect brain-tissue BDNF levels (Klein et al., 2011). In the present work, we observed that the antidepressive-like behavioral effects of AE-PG plus CIT are associated with increased hippocampal BDNF levels, but not with BDNF in plasma. It is important to mention that because BDNF is synthesized in multiple tissues throughout the body, including muscle cells, thymus, or cells of the immune system, such as B- or T-cell and monocytes, among others (Donovan et al., 2000; Nakahashi et al., 2000; Cefis et al., 2022; Matthews et al., 2009), blood/serum circulating BDNF levels may not reflect changes in neurons (Lanz et al., 2012). Thus, our result could suggest that the antidepressive-like effect of AE-PG plus CIT can be exerted through the participation of the hippocampal BDNF.

Synaptophysin protein expression increases in the hippocampus in response to AE-PG and its combination with CIT at non-effective doses. Similar effects are observed after the treatment with several types of antidepressants including escitalopram (Seo et al., 2014) and it has been proposed that this is a mechanism involved in the synaptogenesis induced by antidepressant drugs. Synaptophysin, a synaptic vesicle protein, is located at the presynaptic terminal (Chang et al., 2021; Valtorta et al., 2004) and participates with other proteins, such as BDNF, in the synaptogenesis process (Valtorta et al., 2004; Seo et al., 2014). In turn, the mBDNF protein was also increased in the hippocampus of rats treated with the combination, but not with the drugs alone. Both synaptophysin (presynaptic marker) and BDNF (postsynaptic marker) could together enhance the synaptogenesis process, acting as a more efficient system to contend against stressful situations and regulating the behavioral response. The precise molecular mechanism, as well as the temporal course, by which the combination of AE-PG plus CIT exerts its effects on spine and the synaptogenesis process requires further examination.

Several reports indicate that polyphenols present in pomegranate may inhibit some enzymes of the cytochrome P450 (CYP) 3A family, affecting drug metabolism (Basheer and Kerem, 2015); however, according to the literature, it seems unlikely that the behavioral and neuroplastic effects observed here are related to a pharmacokinetic interaction. In line with this idea, citalopram is the antidepressant that interacts less with other drugs prescribed for medical complications (Brosen and Naranjo, 2001) and is mainly metabolized by CYP2C19, 3A4 and 2D6 (Mrazek et al., 2011). Regarding polyphenols, they are known to be metabolized mainly through the intestinal microbiota (Duda-Chodak et al., 2015); the main route for ellagitannins (Garcñ-Munos and Vaillant, 2014). In addition, polyphenols present in AE-PG inhibit the activity of the CYP3A4 enzyme (Basheer and Kerem, 2015), and the ellagic acid is known to have an inhibitory potential on the CYP1A1 and CYP2E1 enzymes (Ahn et al., 1996). From these data it is unlikely that a pharmacokinetic interaction may modify their bioavailability, promoting a drug-drug interaction. Specific experiments are warranted. An advantage of this combination, based on the behavioral response observed and the literature, is that the inhibitory effect of the polyphenols contained in the AE-PG do not interfere with the metabolism of citalopram, so their combined use could be safe.

In conclusion, the results of this study support the fact that the AE-PG plus CIT combination induces antidepressant-like effects involving the increase of dendritic complexity by increasing the number of dendritic spines, primarily the thin spines category and through the participation of the hippocampal BDNF.

## Data Availability

The original contributions presented in the study are included in the article/supplementary material, further inquiries can be directed to the corresponding author.
